# Trans-omics analyses revealed differences in hormonal and nutritional status between wild and cultured female Japanese eel (*Anguilla japonica*)

**DOI:** 10.1371/journal.pone.0209063

**Published:** 2019-05-09

**Authors:** Masato Higuchi, Miyuki Mekuchi, Takeshi Hano, Hitoshi Imaizumi

**Affiliations:** 1 Shibushi Station, National Research Institute of Aquaculture, Fishery Research and Education Agency, Shibushi-cho, Shibushi, Japan; 2 National Research Institute of Fishery Science, Fishery Research and Education Agency, Fukuura, Kanazawa-ku, Yokohama, Japan; 3 National Research Institute of Fisheries and Environmental of Inland Sea, Fishery Research and Education Agency, Hatsukaichi, Hiroshima, Japan; Shanghai Ocean University, CHINA

## Abstract

Long-term stock decline in the Japanese eel (*Anguilla japonica*) is a serious issue. To reduce natural resource utilization in Japan, artificial hormonal induction of maturation and fertilization in the Japanese eel has been intensively studied. Recent experiment on feminized (by feeding a commercial diet containing estradiol-17β for first half year) cultured female eels have shown ovulation problems, which is seldom observed in captured wild female eels. Therefore, the aim of this study is to try to investigate causes of ovulation problem frequently seen in cultured female eels by comparative trans-omics analyses. The omics data showed low growth hormone and luteinizing hormone transcription levels in the brain and low sex hormone–binding globulin transcription levels in the liver of the cultured female eels. In addition, it was found that high accumulation of glucose-6-phosphate and, maltose in the liver of the cultured female eel. It was also found that docosahexaenoic (DHA) acid, eicosapentaenoic acid (EPA) and arachidonic acid (ARA) ratios in cultured female eels were quite different from wild female eels. The data suggested that ovulation problem in cultured female eels was possibly resulted from prolonged intake of a high-carbohydrate diet and/or suboptimal DHA/EPA/ARA ratios in a diet.

## Introduction

The Japanese eel (*Anguilla japonica*) is an important species for inland aquaculture in Japan because of its high economic value. The seed used in its cultivation is taken from wild glass eels captured from estuaries. However, as the glass eel arrival has been drastically dropped since 1970s, eel seed depletion has become a serious problem not only in Japan but also in other East Asian countries. To reduce natural resource utilization in Japan, artificial breeding techniques of eels have been studied, and rearing methods of eel leptocephali have been developed [1].

Cultured female and male eels do not mature sexually in captive conditions. Therefore, weekly injection of salmon pituitary extract (SPE) is administrated to captured wild female eels, acclimated to seawater, to induce oocyte maturation [2]. When the body weight of female increased by 10% from the start of weekly injection of SPE (after 10–15 injections of SPE) 17α-hydroxyprogesterone is injected to induce final oocyte maturation and ovulation [3]. To induce maturation of male eels, recombinant eel luteinizing hormone is used (human chorionic gonadotropin was used in the past [4]).

The matured female and male eels are then transferred to a tank. When water temperature is raised from 20°C to 22°C, spawning takes place early in the morning. The Japanese eel has gymnovarian–type ovaries, which lack a part of the ovarian capsule; therefore, ovulated eggs are discharged into the abdominal cavity once and then spawned through the genital pore [5]. About 0.3–1.0 million fertilized eggs can be collected from one wild female eel by using this method.

**Fig 1:**
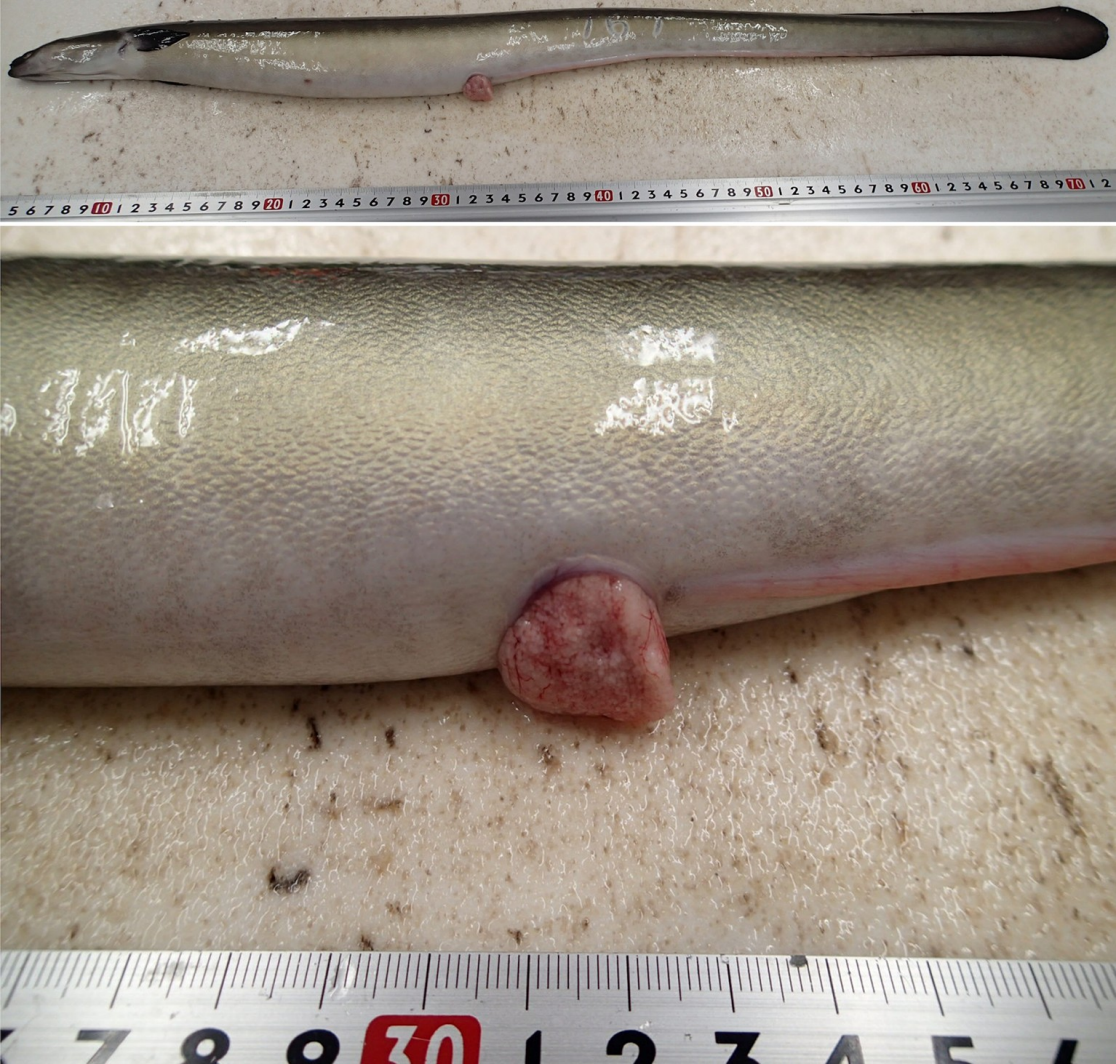
Clogged oviduct after hormone-induced spawning in cultured eel.

In the recent year, feminized (by feeding a commercial diet containing estradiol-17β for the first half year) cultured female eels have been used on an experimental basis to reduce natural resource utilization. It has become possible to obtain fertilized eggs from cultured female eels. However, it has been observed that in cultured female eels, the genital pore is clogged with ovarian fragment during artificial spawning (Fig 1), therefore, normal release of eggs is severely disrupted. Anatomical observations have shown that many unovulated eggs remained in the ovary and ovarian fragment hypertrophy in cultured female eels after artificial spawning, compared to fully ovulated eggs in wild female eels. In 2015–2016, in 28.9% (39/135) of cultured female eels, the genital pore was clogged and more than 50-g eggs (approximately 2000 eggs/g) remained in the abdomen while in 1.6% (1/61) of wild female eels, the genital pore was clogged after artificial spawning. Furthermore, the average number of spawned eggs and the quality of fertilized eggs (e.g. fertilization, hatching, larval survival, and larval malformation rates) in cultured female eels were lower than wild female eels. It has been reported that in cultured female eels, serum estradiol-17β and 11-ketosterone levels and transcription levels of receptors for luteinizing hormone and follicle-stimulating hormone were increased during late stage of artificial oocyte maturation [6, 7]. Lipid contents of liver also increased during late stage of artificial oocyte maturation, while lipid contents of ovary increased until mid-vitellogenic stage, and the decreased toward ovulation in both cultured and wild female eels [8]. These findings indicated that in cultured female eel, there are problems with ovulation rather than oocyte maturation, but the reason is unclear. Hence, it is hypothesized that the physiological status of the eels just before hormone injection was the reason for this difference since artificial induction of oocyte maturation method was common between cultured and wild female eels.

In this study, we examined the differences between cultured and wild female eels using combined transcriptomic and metabolomic analyses, also called trans-omics analysis. New omics technologies have already been applied to aquaculture study [9]. In comparison with wild female eels, cultured female eels showed quite different hormonal and nutritional status just before the beginning of induction of artificial oocyte maturation. These differences that will be clarified from this study would serve as keys to improving ovulation problems in cultured female eels.

## Materials and methods

### Ethics statement

All experiments were conducted in accordance with the institutional procedures of the National Research Institute of Aquaculture guidelines. The protocol was approved by the committee on the National Research Institute of Aquaculture (Permission No. 29016). All surgery performed under 2-phenoxyethanol anesthesia, and all efforts were made to minimize suffering.

### Animals

We purchased 20 wild female eels (from Lake Jinzai, Japan: 35°19^’^ N, 132°40^’^ E; body weight [BW]: 500–1000 g) from Mitani eel wholesaler, the local eel supplier in Shimane prefecture, on September 29, 2016. Acclimation to seawater was followed by usual procedure [10]. Briefly, the eels were stocked in a 500-L tank, into which seawater (26–27°C) was poured. After 36 h, the salinity was 33 ‰. Afterwards, 5 eels were randomly selected and transferred to a 2-kL semi-intensive tank filled with seawater, and the water temperature was gradually decreased from 26°C to 20°C (at the rate of 1.5°C /day). At the end of temperature adjustment, eels were then kept in the tank for 14 days without feeding and without light until the sampling date.

To obtain wild male eel, 20 small-size wild eels (BW: 180–250 g) were purchased from the same eel supplier. The eels were acclimated to seawater and water temperature of 20°C as mentioned earlier and reared for 14 days without feeding and without light until the sampling date.

For feminization experiment, 1000 juvenile grass eels were purchased from Oosumi fishery cooperative, the commercial eel supplier in Kagoshima prefecture on February 1, 2013. After acclimation to commercial fish feed, the eels were fed on a diet with E2 (10 mg/kg diet) [11] every day until July 29, 2013, after which the eels were fed modified commercial feed without E2 supplementation (Table 1) on every Monday, Wednesday, and Friday. The feeding ratio was periodically adjusted on the basis of appetite. The eels were reared in fresh water at natural water temperature until September 29, 2016. Then 5 feminized cultured female eels (BW: 800–900 g) were randomly selected and transferred into a 500-L tank and same acclimation treatments as in the above mentioned were carried out, and they were kept in the tanks for 14 days without feeding and without light until the sampling date.

**Table 1:** Diet formulation for cultured female eels.

Component (Manufacturer, Country)	(g)
Commercial feed for eels (Marubeni-Nisshin, Japan)	600
-Fish meal (75%)	
-Potato starch (23%)	
-Mineral premix* (2%)	
Shrimp and crab meal (Bio-Kagaku, Japan)	9
Vitamin premix (Marubeni-Nisshin, Japan)	30
Cod liver oil (Kanematsu-Shintoa, Japan)	48
Water	780
Total	1467

*The mineral premix mainly consists of CaCO_3_, NaCl, and Ca(H_2_PO_4_)_2_.

### Tissue collection

The cultured (n = 5) and wild (n = 5) female eels were euthanized with 0.5% 2-phenoxyethanol. After total length and body weight measurements, the brain, liver, ovaries, and blood were sampled from each eel; Table 2 shows the data collected. The whole brain and 5-mm^3^ pieces of the liver and ovaries were immediately placed in RNAlater solution (Ambion, Austin, TX, USA) and stored at −25°C. The liver and ovary samples were then weighed and sliced into approximately 10-mg pieces, frozen in liquid nitrogen, and stored at −80°C until analysis. We also fixed 5-mm^3^ pieces of the ovary samples in 10% neutral phosphate-buffered formalin and stored at 4°C until analysis. The remaining liver and ovary samples were stored at −80°C. The blood samples were stored for 3h at 4°C and then centrifuged at 1300 × g for 10 min at 4°C. Subsequently, the serum was collected, aliquoted, and then stored at −80°C until analysis. Twenty small size eels were euthanized with 0.5% 2-phenoxyethanol, and then gender was determined by morphological observation of the gonads. Five eels were male, and the rest were female. Brains of wild male eel were immediately placed in RNAlater solution and stored at −25°C. To avoid effects of circadian rhythm, tissue collection was done at the same hour (10:00–12:00).

**Table 2:** The collected data from cultured and wild female eels at the day of sampling.

	Cultured	Wild
Total length (cm)	80.8 ± 1.1	75.4 ± 1.8
Body weight (g)	841.8 ± 20.3	766.2 ± 45.5
Condition factor	0.159 ± 0.006	0.179 ± 0.005*
Liver weight (g)	10.7 ± 1.2	8.5 ± 0.2
HSI	1.3 ± 0.1	1.1 ± 0.1
Gonad weight (g)	9.6 ± 0.9	12.4 ± 0.6*
GSI	1.1 ± 0.1	1.6 ± 0.1*

Values are expressed as the mean ± standard error of mean (SEM) of 5 samples

Condition factor = 100 × weight(g)/length(cm)^3

Hepatosomatic index: HSI = [liver weight/body weight] × 100.

Gonadosomatic index: GSI = [ovary weight/body weight] × 100.

* P < 0.05.

### Transcriptomic analysis

Total RNA was extracted using the RNeasy Lipid Tissue Mini Kit (Qiagen, GmbH, Hilden, Germany) according to the manufacturer’s instruction. The RNA quality of the whole brain and the liver were evaluated on the basis of the proportion of ribosomal RNA (rRNA) in them using the Agilent 2100 Bioanalyzer Nano 6000 Kit (Agilent technologies, Palo Alto, CA, USA). The average RNA integrity numbers (RINs) of the whole brain and the liver were 9.0 and 7.0, respectively. However, since several reports have shown that due to unusual RNA composition, RINs of ovary samples in primary growth stages cannot be calculated using the RIN algorithm [12], we did not analyze the RINs of the ovary samples. In all, 4 total RNA (500 ng) samples (no.1 -no. 4) from each eel group (cultured female brain, liver, and ovary; wild female brain, liver, and ovary; wild male brain) were processed using the TruSeq RNA Sample Preparation v2 Kit (illimuna, San Diego, CA, USA) according to the manufacturer’s instructions. cDNA libraries with barcodes were constructed in order to enable multiplexing of pools, and sequenced the libraries using the illumina Hi-Seq 4000 platform equipped with a 50-base pair (bp) single-end module. The sequence data was deposited into DDBJ sequence read archive. The accession numbers are DRA007019 (brain), DRA007045 (liver), and DRA007046 (ovary), respectively. For postsequencing, raw reads were filtered by removing low-quality reads (quality value [QV] < 20). Next, we performed read-mapping analysis using CLC Genomics Workbench 8.1 (https://www.qiagenbioinformatics.com/) using the predicted protein-coding genes in the Japanese eel genome as a reference [13], and the expression value was calculated using the total number of reads mapped to the genes. Statistical analyses included the digital gene expression (DGE) test of CLC Genomics Workbench 8.1, and differences at *p < 0*.*05* were considered statistically significant.

### Quantitative polymerase chain reaction

After priming with a random hexamer, first-strand cDNA was synthesized from 1 μg of total RNA using the Omniscript RT Kit (Qiagen GmbH, Dusseldorf, Germany). Five total RNA samples were obtained from each eel group (brain and liver from cultured female eels; brain and liver from wild female eels; brain from wild male eels). With four of the samples (numbers 1–4), the same source of total RNA was used for both the transcriptomic and the quantitative polymerase chain reaction (RT-qPCR) analyses. The fifth total RNA samples (no. 5) from each eel group was used only for RT-qPCR. Primers and probes were designed in reference to the Japanese eel genome data mentioned before [13]; see S1 Table for primer and probe sequences. Information about selected genes for RT-qPCR analyses, their functionalities and tissue are listed in Table 3. The PCR mixture comprised each primer pair (0.5 μM) and probe (0.2 μM), and the FastStart Essential DNA Probes Master (2x) (Roche Diagnostics, Switzerland). The data was analyzed using the CFX96 Touch Real-time PCR Detection System (Bio-Rad, Hercules, CA, USA). The expression levels of messenger RNA (mRNA) in the samples were normalized to 60S acidic ribosomal protein P0 (*rplp0*) mRNA expression and calculated using the 2^-ΔΔCt^ method. PCR efficiencies of each primer pair were calculated from the slope of each standard curve with the equation and listed in S1 Table.

**Table 3:** Selected genes, their functionalities, and used tissue for RT-qPCR analyses.

Gene	Function	Tissue
3-oxo-5-beta-steroid 4-dehydrogenase	An enzyme that catalyzes the reduction of progesterone, androstenedione, 17-alpha-hydroxyprogesterone and testosterone to 5-beta-reduced metabolites.	Liver
cytochrome P450 1A9	An enzyme that catalyzes 16α hydroxyprogesterone to 6β hydroxyprogesterone. Also play as drug metabolizing enzyme.	Liver
sex hormone-binding globulin	Testosterone and 17 beta-estradiol transport protein.	Liver
follicle stimulating hormone	Gonadotropin, regulates pubertal maturation and reproductive processes.	Brain
luteinizing hormone	This hormone surge triggers ovulation.	Brain
growth hormone	Peptide hormone, play an important role in growth control, and participates in gonadal steroidogenesis, gametogenesis and ovulation.	Brain
somatolactin	May play role in ion regulation and reproduction	Brain
cytochrome P450 19A1	An enzyme responsible for the biosynthesis of estrogens.	Brain
protein jumonji	A member of the alpha-ketoglutarate-dependent hydroxylase superfamily.	Brain

### Metabolomics analysis

The chemicals we used in the gas chromatography-mass spectrometry (GC-MS)-based metabolomics study were as follows: methoxyamine hydrochloride (Sigma Aldrich, St. Louis, MO, USA)); pyridine (Kanto Kagaku Chemical, Japan); pesticide-analytical-grade chloroform, HPLC-analytical-grade methanol, 2-propanol, acetonitrile, and ultrapure water (Wako Chemical, Japan); and N-methyl-N-(trimethylsilyl)-trifluoroacetamide (MSTFA) (GL Science Inc., Japan). Ribitol solution (Wako, Japan) in MilliQ water (200 mg/L) was used as an internal standard. Also, d27-Myristic acid (400 mg/L, Sigma Aldrich) was prepared in methanol in order to lock the retention time as described later.

To perform metabolomics on the liver and ovaries [14], we combined 1 mL of solvent mixture (CH_3_Cl:CH_3_OH:H_2_O, 1:2.5:1, v/v) and 60 μL of ribitol solution (10 mg ribitol dissolved in 100 mL water) with a tissue sample in a 2-mL microcentrifuge tube. Zirconia balls (1 mm in diameter) were added to the tube, and the sample was subjected to a sample crusher system (Fast Prep 24 Instrument, Funakoshi, Tokyo, Japan) for 1 min. The sample was then shaken for 30 min at 37°C and centrifuged at 16,000 × *g* for 5 min at 4°C. The supernatant (900 μL) was mixed with 400 μL of water and centrifuged again at 16,000 × *g* for 5 min, resulting in the formation of two phases: water phase (supernatant) and organic phase (bottom layer). The supernatant (800 μL) was mixed with 50 μL of d27-myristic acid solution and transferred to a 1.5-mL Eppendorf tube with a pierced cap. In addition, 100 μL of organic phase was collected and mixed with 50 μL of d27-myristic acid solution.

To prepare a serum, 50 μL of each sample were quenched by using 0.25 ml of a solvent mixture (acetonitrile:2-propanol: ultrapure water, 3:3:2, v/v) and mixed with 60 μL of ribitol and 50 μL of d27-myristic acid solution. The resulting extraction mixture of liver, ovaries, and serum was completely dried in a vacuum centrifuge drier (CVE-3000, Tokyo Rikakikai, Japan). For all samples, except the organic phase extracts of liver and ovaries, we performed two-step derivatization: (i) oximation and (ii) silylation. For oximation, 100 μL of methoxyamine hydrochloride was added in pyridine (20 mg/L) to the samples, and incubated the mixture for 90 min at 30°C. Thereafter, 50 μL of MSTFA was added to the samples for silylation, followed by incubation for 30 min at 37°C. The organic phase extracts were dissolved in 100 μL of pyridine followed by silylation with 50 μL of MSTA for 30 min at 37°C.

GC separation of the metabolites was performed using an Agilent 6890N gas chromatograph (Agilent technologies, Japan), equipped with a quadrupole mass spectrometer (Agilent 5975) and a DB-5ms column (i.d. 0.25 mm x 30 m, 0.25 μm thickness, 10 m DG, Agilent technologies). The samples (1 μL each) were injected in split mode (10:1, v/v); the temperatures of the injector, transfer line, and ion source were 250°C, 275°C, and 280°C, respectively. The helium gas flow rate was adjusted to 1.1 mL/min for a d27-myristic acid retention time of 16.727 min, and the oven temperature program was set as follows: 60°C for 1 min, increased at a rate of 10°C/min to 325°C, and then held at 325°C for 10 min.

For data processing, we performed ion chromatographic deconvolution using the Automated Mass Spectral Deconvolution and Identification System (AMDIS, ver.2.7, Agilent, CA, USA). Metabolites were identified by their mass spectral patterns and retention times using the Fiehn Library (Agilent) as implemented in MSD ChemStation (Agilent). 61 metabolites were successfully identified (51 for the water phase, and 10 for the organic phase), 58 metabolites (47 for the water phase, and 11 for the organic phase) and 65 metabolites in the tissue samples of the liver, ovaries and serum, respectively.

### Serum lipids quantification

Serum cholesterol, triglyceride and phospholipids levels were measured using the Wako series test kit (Wako, Japan) according to the manufacturer’s microplate instructions.

### Serum and ovarian steroids quantification

Liquid chromatography-tandem mass spectrometry (LC-MS/MS) was used to assay serum estrone, E2, androstenedione, testosterone (T), 5α-dihydrotestosterone (5α-DHT), 11-ketotestosterone (11-KT), progesterone and 17-hydroxyprogesterone levels and ovarian E2 and T levels. For detailed procedures refer to other studies [15].

### TUNEL assay

Results of transcriptomic analysis of the ovary indicated that apoptosis occurs in cultured female eel ovaries due to high expression of apoptosis-associated genes, such as small nuclear ribonucleoprotein F, tumor necrosis factor receptor superfamily member 19, cytochrome c, and granzyme K. Therefore, the terminal deoxynuculeotidyl transferase triphosphate (dUTP) nick end labeling (TUNEL) was used to confirm that cell death occurs through apoptosis. Ovary samples were fixed in 10% neutral phosphate-buffered formalin, embedded in paraffin wax and sectioned at 5-μm thickness. Then TUNEL assay was performed using ApopTag plus *in situ* apoptosis detection kit (Merck Millipore, UK) and hematoxylin counterstaining according to the manufacturer’s instructions.

### Statistical analysis

We used the *t*-test to measure statistical differences in the gonad somatic index (GSI), hepatosomatic index (HSI), serum lipids levels, serum steroid levels, ovarian steroid levels, and quantitative PCR analyses.

For qPCR of the brain samples of cultured female, wild female, and wild male, statistical differences were determined by one-way ANOVA, followed by Tukey’s test.

For metabolite analysis, for water phase (liver and ovary samples) and the serum, we normalized unique masses of each metabolite peak using the unique mass of the ribitol peak (m/z 217). For the organic phase of the liver and ovary samples, we performed normalization using the unique mass of the d-27 myristic acid peak (m/z 312). To identify key metabolites that help differentiate between cultured and wild female eels, we performed orthogonal partial least-square discriminant analysis (OPLS-DA) followed by the S-plot using SIMCA 14.0 (Umetrics, Umea, Sweden). Pareto (Par) scaling of the normalized value was selected before fitting. MS-DIAL was used to find the differences in metabolites between two experimental eel groups using the *t*-tests for parametric data and Welch’s *t*-tests for nonparametric data at *p < 0*.*05* [16].

## Results

### Transcriptomic assembly

Transcriptomic analysis was carried out to evaluate the differences in gene expression patterns between wild and cultured female eels. The cDNA libraries constructed from eel liver, ovary, and brain samples were sequenced using Illumina Hi-Seq 4000. From each individual sample, approximately 20 million sequence reads were obtained on average, and approximately 70% of the reads were mapped to the reference sequences. mRNA transcripts satisfied the *p < 0*.*05* criterion of the Gaussian-based test, and a fold change > 2.0. For further analysis, normalized expression values > 100 in the higher-expressed eel group were used (See S2–S5 Tables) for a list of screened mRNA transcripts.

### Gene expression comparison

**Fig 2:**
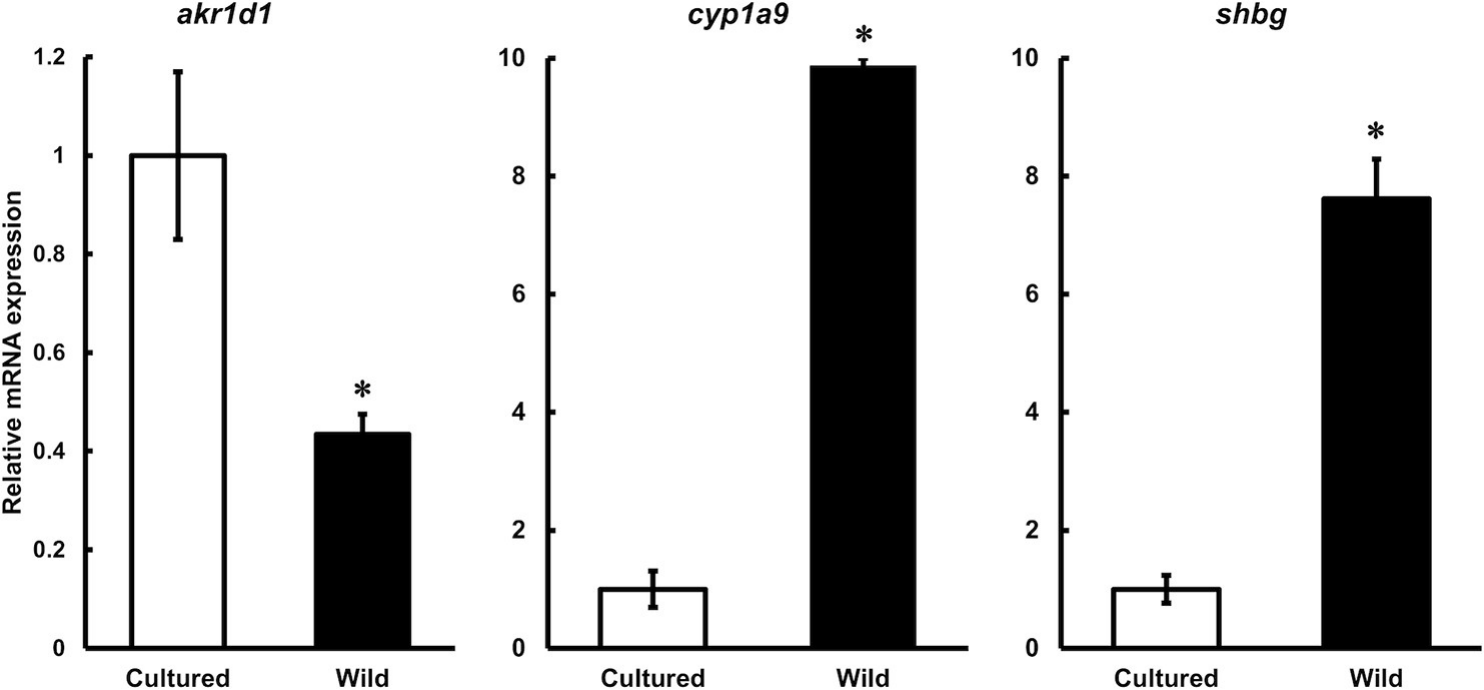
RT-qPCR analysis of liver samples. RT-qPCR analysis of 3-oxo-5-beta-steroid 4-dehydrogenase (*akr1d1)*, cytochrome P450 1A9 *(cyp1a9)*, and sex hormone-binding globulin (*shbg)* mRNA expression in cultured and wild female eel liver samples Relative mRNA expression is normalized against 60S acidic ribosomal protein P0 *(rplp0)* mRNA expression. Each vertical bar represents the mean ± SEM of 5 samples. *P < 0.*01*.

On the basis of the results of RNA-seq (S2–S5 Tables), we performed validation and further analysis using real-time quantitative PCR (RT-qPCR). In the liver samples, we examined mRNA expression of 3-oxo-5-beta-steroid 4-dehydrogenase (*akr1d1*), cytochrome P450 1A9 (*cyp1a9*), and sex hormone-binding globulin (*shbg*) because these genes are related to steroidogenesis and steroid transport. We observed the mRNA expression level of *akr1d1* was 1.3-fold higher in cultured female eels compared to wild female eels, while *cyp1a9* and *shbg* mRNA levels were 8.9-fold and 6.6-fold higher, respectively, in wild female eels compared to cultured female eels (Fig 2).

**Fig 3:**
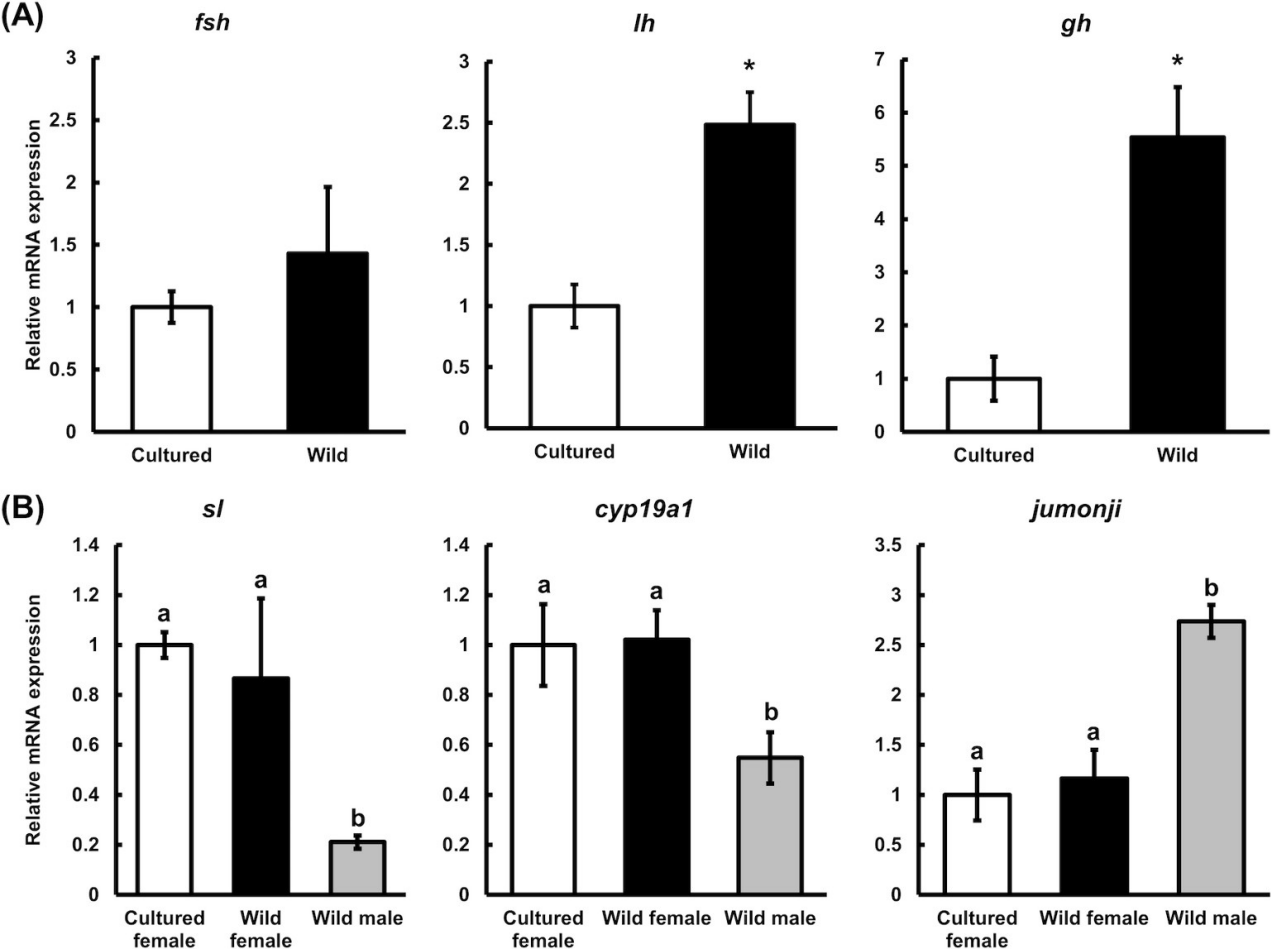
RT-qPCR analysis of eel brain samples. (A) RT-qPCR analysis of follicle stimulating hormone *(fsh)*, luteinizing hormone *(lh)*, and growth hormone (*gh)* in cultured and wild female eel brain samples. Relative mRNA expression is normalized against 60S acidic ribosomal protein P0 (*rplp0*) mRNA expression. Each vertical bar represents the mean ± SEM of 5 samples. *P < *0*.*05*. (B) RT-qPCR analysis of somatolactin (*sl)*, cytochrome P450 19A1 (*cyp19a1)*, and protein jumonji (*jumonji)* mRNA expression in the brain samples of cultured female, wild female, and wild male eels. Relative mRNA expression is normalized against 60S acidic ribosomal protein P0 (*rplp0*) mRNA expression. Each vertical bar represents the mean ± SEM of 5 samples. *a and b* indicate significant differences (*P*< 0.05).

In the brain samples, we analyzed the expression levels of *follicle-stimulating hormone* (*fsh*), *luteinizing hormone* (*lh*), and *growth hormone* (*gh*) because these peptide hormones are closely related to reproduction. Although no significant difference was observed in the *fsh* transcription levels between wild and cultured female eels (same as in the case of RNA-seq), the *lh* and *gh* transcription levels in wild female eels were 1.5-fold and 4.5-fold higher, respectively, than in cultured female eels (Fig 3A).

In cultured female eels, due to lower *shbg* transcription levels, we suspected hyperandrogenism and masculinization. To confirm this, marker genes showing sex-dependent expression patterns from S5 Table. *somatolactin* (*sl*) and *cytochrome P450 19A1* (*cyp19a1*) were selected as female highly expressed genes, and *protein jumonji* (*jumonji*) was selected as the male highly expressed gene. No significant differences were observed between wild and cultured female eels in the selected gene expression levels; however, there were significant differences between female and male eels (Fig 3B). Results of transcriptomic analysis showed that the expression levels of the four JUMONJI family genes are different between wild female and wild male in the brains. Amino acid sequences of four JUMONJI family genes contain ARID, jmjC and/or jmjN domains, which characterize JUMONJI; however, the sequences show some differences from one another. Primers and probe were designed for the no. 6 of in the S5 Table because only no. 6 contains all three ARID, jmjC, and jmjN domains. The mRNA expression pattern obtained from qPCR was the same as that obtained from RNA-seq data.

### Serum lipids and steroids, and ovarian steroid levels

Serum cholesterol, triglyceride, and phospholipids levels were measured (Table 4). There were no significant differences in cholesterol and phospholipid levels between wild and cultured female eels, while serum triglyceride levels of cultured female eels were significantly higher than those of wild female eels.

**Table 4:** Serum lipid, steroid levels, and ovarian steroid levels in eels.

	Cultured	Wild
**Serum lipid levels**		
Cholesterol (mg/dL)	315.4 ± 35.1	303.4 ± 29.1
Triglyceride (mg/dL)	971.4 ± 145.2	543.0 ± 55.0*
Phospholipids (mg/dL)	659.7 ± 58.3	731.2 ± 42.5
**Serum steroid levels**		
Estradiol-17β (pg/mL)	99.5 ± 28.5	85.6 ± 26.6
Androstenedione (pg/mL)	12.1 ± 3.5	12.2 ± 4.8
Testosterone (ng/mL)	0.63 ± 0.2	1.03 ± 0.5
5α-Dihydrotestosterone (pg/mL)	23.2 ± 4.1	37.8 ± 14.1
11-Ketotestosterone (ng/mL)	0.64 ± 0.2	1.14 ± 0.2
17-Hydroxyprogesterone (pg/mL)	76.1 ± 38.4	43.1 ± 6.2
**Ovarian steroid levels**		
Estradiol-17β (pg/g-wet)	68.7 ± 17.9	62.1 ± 23.5
Testosterone (pg/g-wet)	129.4 ± 29.5	223.7 ± 77.3

Values are expressed as the mean ± SEM (n = 5).

* P < 0.05.

LC-MS/MS was used to assay total serum estrone, E2, androstenedione, T, 5α-DHT, 11-KT, progesterone and 17-hydroxyprogesterone levels, and ovarian E2 and T levels (listed in Table 4). The estrone and progesterone levels were under the lower limit of quantitation (1 pg/mL and 2 pg/mL, respectively), and therefore were omitted. Serum T and 11-KT levels were lower in cultured female eels than in wild female eels, although no significant differences were observed.

Ovarian T levels were also lower in cultured female eels, although, again, there were no significant differences.

### Metabolomics

**Fig 4:**
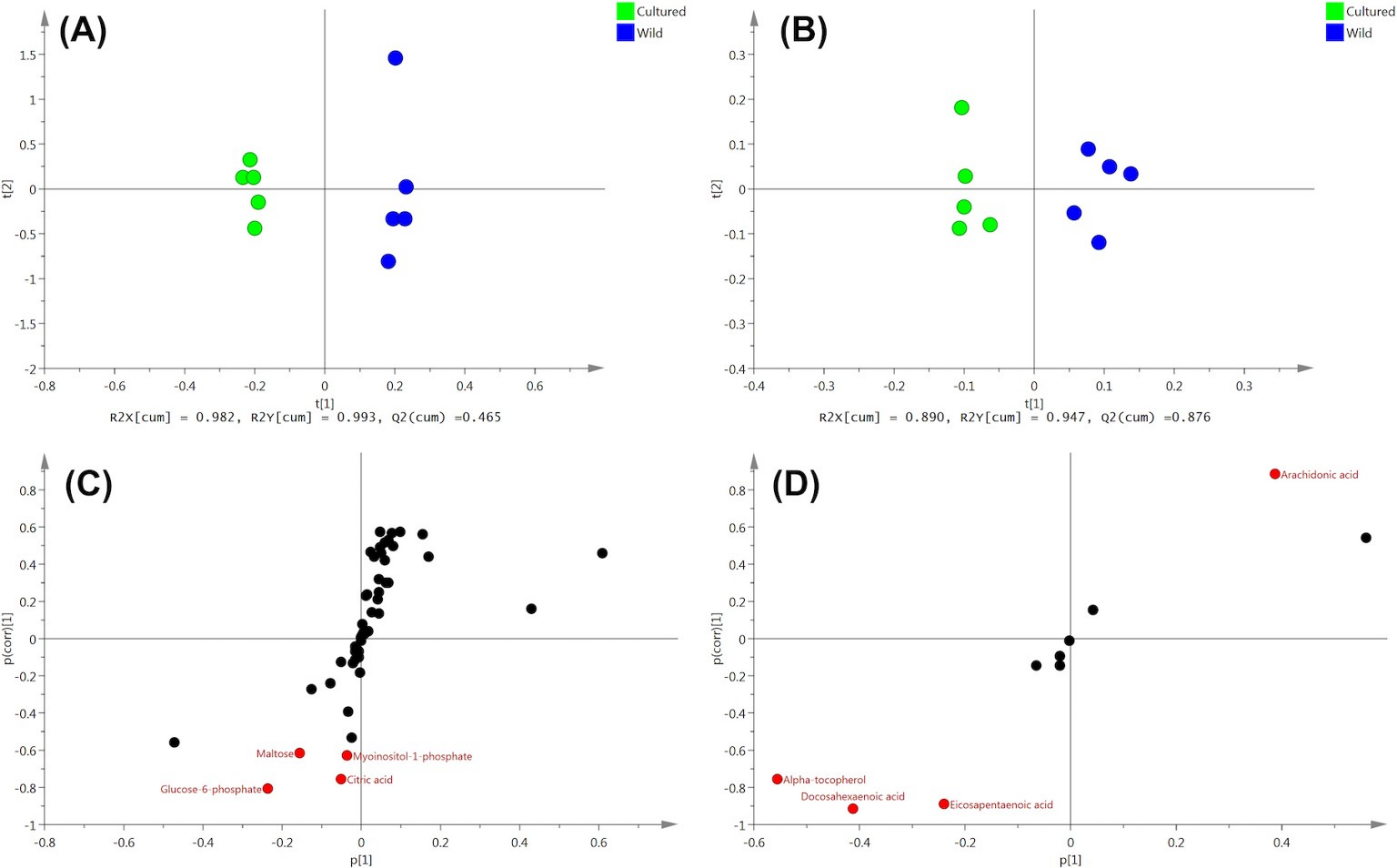
OPLS-DA of liver metabolites obtained from cultured and wild female eels. (A) water phase fraction and (B) organic phase fraction. S-plot generated from OPLS-DA of (C) water phase fraction and (D) organic phase fraction.

**Fig 5:**
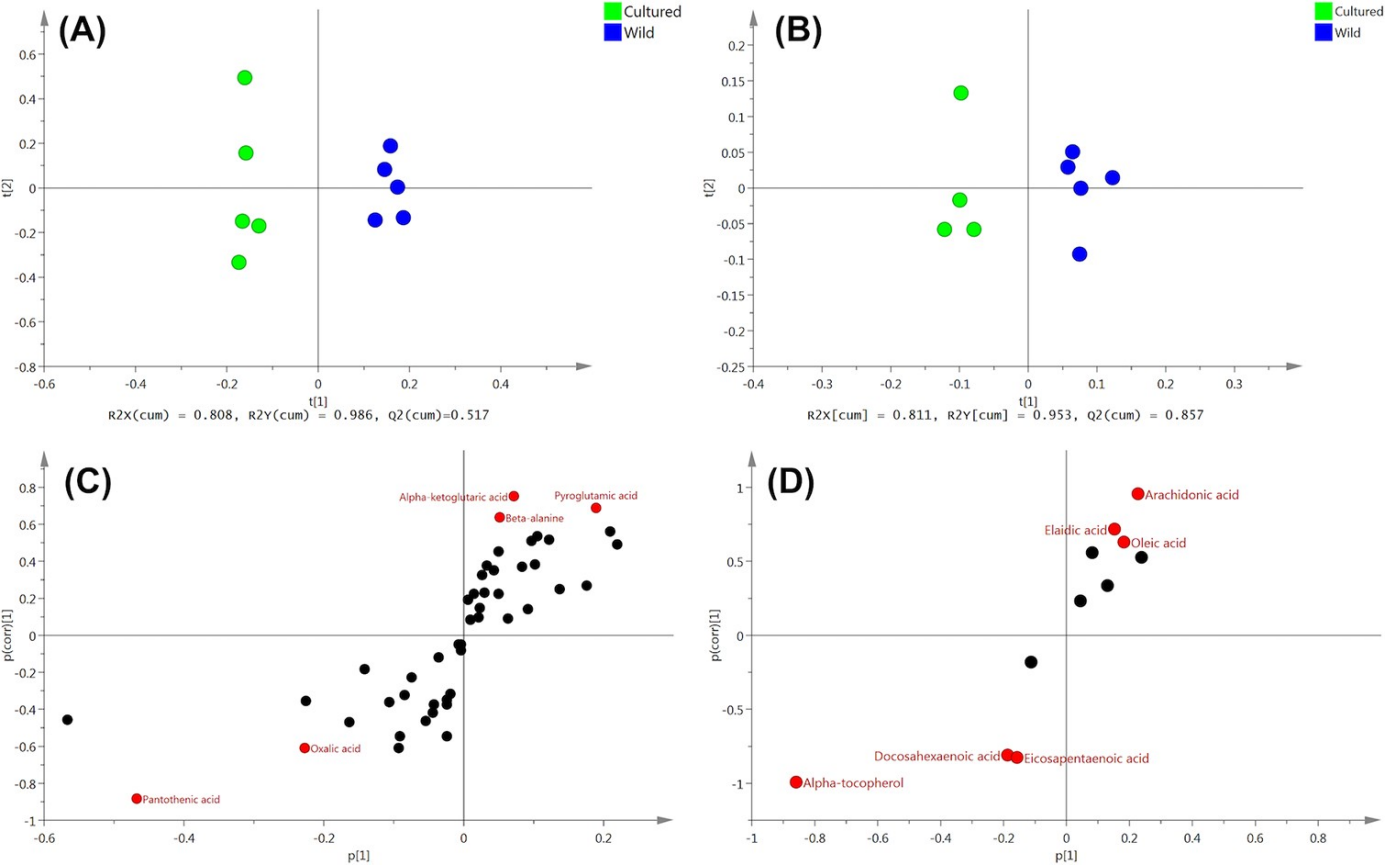
OPLS-DA of ovary metabolites obtained from cultured and wild female eels. (A) water phase fraction. (B) organic phase fraction. S-plot generated from OPLS-DA of (C) water phase fraction and (D) organic phase fraction.

**Fig 6:**
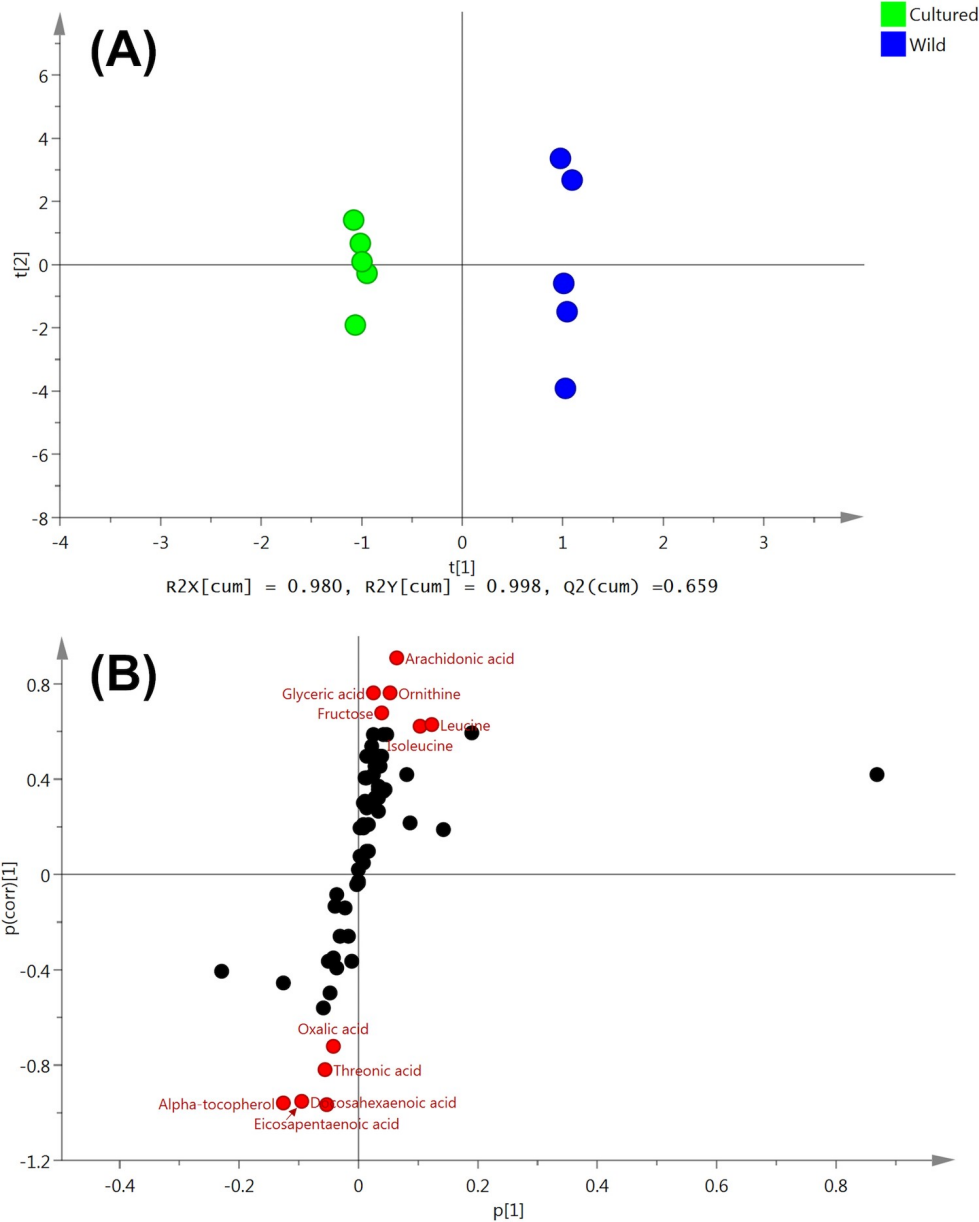
OPLS-DA of serum metabolites obtained from cultured and wild female eels. (A) OPLS-DA of serum metabolites obtained from cultured and wild eels. (B) S-plot generated from OPLS-DA.

For metabolomic analysis, we performed OPLS-DA followed by the S-plot in order to estimate the crucial metabolites contributing to differences between cultured and wild female eels. For detailed procedures refer to other study [17]. For all tissues, the predictive component t[1] successfully separated the two eel groups. (Figs 4A, 4B, 5A, 5B and 6A). Candidate biomarkers, with an absolute p(corr) value of greater than 0.6, were selected [18] (Figs 4C, 4D, 5C, 5D and 6B). R2 and Q2 were used to evaluate the model. R2 represents the model’s goodness. R2X is the fraction of the variation of the X variables, and R2Y is the fraction of the variation of Y variables explained by the model. Q2 represents the model’s goodness of prediction and also an estimate of the model’s predictive ability. Q2 is calculated by cross-validation. The higher the values of R2 and Q2, the better is the model’s predictive ability.

In the liver, 8 metabolites were identified as candidate biomarkers; wild female eels showed high levels of 1, whereas cultured female eels showed high levels of 7 (Table 5). In cultured female eels, sugars (glucose-6-phosphate and maltose) showed a about 5-fold increase, which may have important implications for energy metabolism disorders.

**Table 5:** Fold change of biomarker candidates between cultured and wild female eel livers.

Metabolites	Fold change (Cultured/Wild)	*P-value*
Arachidonic acid	0.22	*
Myoinositol-1-phosphate	1.34	
Docosahexaenoic acid	1.90	**
Citric acid	1.91	**
Eicosapentaenoic acid	1.97	**
Glucose-6-phosphate	4.96	**
Maltose	5.66	
Alpha-tocopherol	9.45	*

* and ** denotes significant difference at *P < 0*.*05* and *P < 0*.*01*, respectively.

In the ovaries, 11 metabolites were identified as candidate biomarkers; wild female eels showed high levels of 6, whereas cultured female eels showed high levels of 5 (Table 6). Pantothenic acid (vitamin B5) was uniquely detected only in the ovaries and increased by 1.8-fold in cultured female eels. Unfortunately, one sample of organic phase extract from cultured female eel ovary was not correctly analyzed by GC/MS, therefore, this sample was omitted from analysis.

**Table 6:** Fold change of biomarker candidates between cultured and wild female eel ovaries.

Metabolites	Fold change (Cultured/Wild)	*P-value*
Arachidonic acid	0.37	**
Pyroglutamic acid	0.43	
Elaidic acid	0.53	*
Alpha-ketoglutaric acid	0.57	*
Oleic acid	0.74	
Beta-alanine	0.76	*
Docosahexaenoic acid	1.50	*
Panthothenic acid	1.81	**
Oxalic acid	1.84	*
Eicosapentaenoic acid	2.03	**
Alpha-tocopherol	5.27	**

* and ** denotes significant difference at *P < 0*.*05* and *P < 0*.*01*, respectively.

In the serum samples, 11 metabolites were identified as candidate biomarkers; wild female eels show high levels of 6, whereas cultured female eels showed high levels of 5 (Table 7). Along with eicosapentaenoic acid (EPA), docosahexaenoic acid (DHA) showed a significant increase in cultured female eels, while arachidonic acid (ARA) was found in high levels in wild female eels. Threonic acid and oxalic acid, which are metabolites of ascorbate, increased in cultured female eels.

**Table 7:** Fold change of biomarker candidates between cultured and wild female eel serum.

Metabolites	Fold change (Cultured/Wild)	*P-value*
Arachidonic acid	0.14	**
Fructose	0.30	
Ornithine	0.45	*
Isoleucine	0.53	
Leucine	0.55	
Glyceric acid	0.58	*
Oxalic acid	1.58	*
Threonic acid	2.64	**
Docosahexaenoic acid	2.81	**
Eicosapentaenoic acid	2.84	**
Alpha-tocopherol	3.18	**

* and ** denotes significant difference at *P < 0*.*05* and *P < 0*.*01*, respectively.

It is noteworthy that EPA, DHA, and α-tocopherol (lipophilic vitamin E) increased in all three tissue samples of cultured female eels, while ARA increased in all three tissue samples of wild female eels. The DHA/EPA/ARA ratios calculated by normalized average peak area (peak areas of each fatty acids/peak area of internal standard/sample weight-dry) by GC/MS analyses in cultured and wild female eels were 10.1/3.2/1.0 and 1.1/0.3/1.0, respectively in the liver; 4.0/1.9/1.0 and 1.0/0.4/1.0, respectively in the ovaries; and 19.5/6.5/1.0 and 1.1/0.3/1.0, respectively, in the serum.

### TUNEL assay

**Fig 7:**
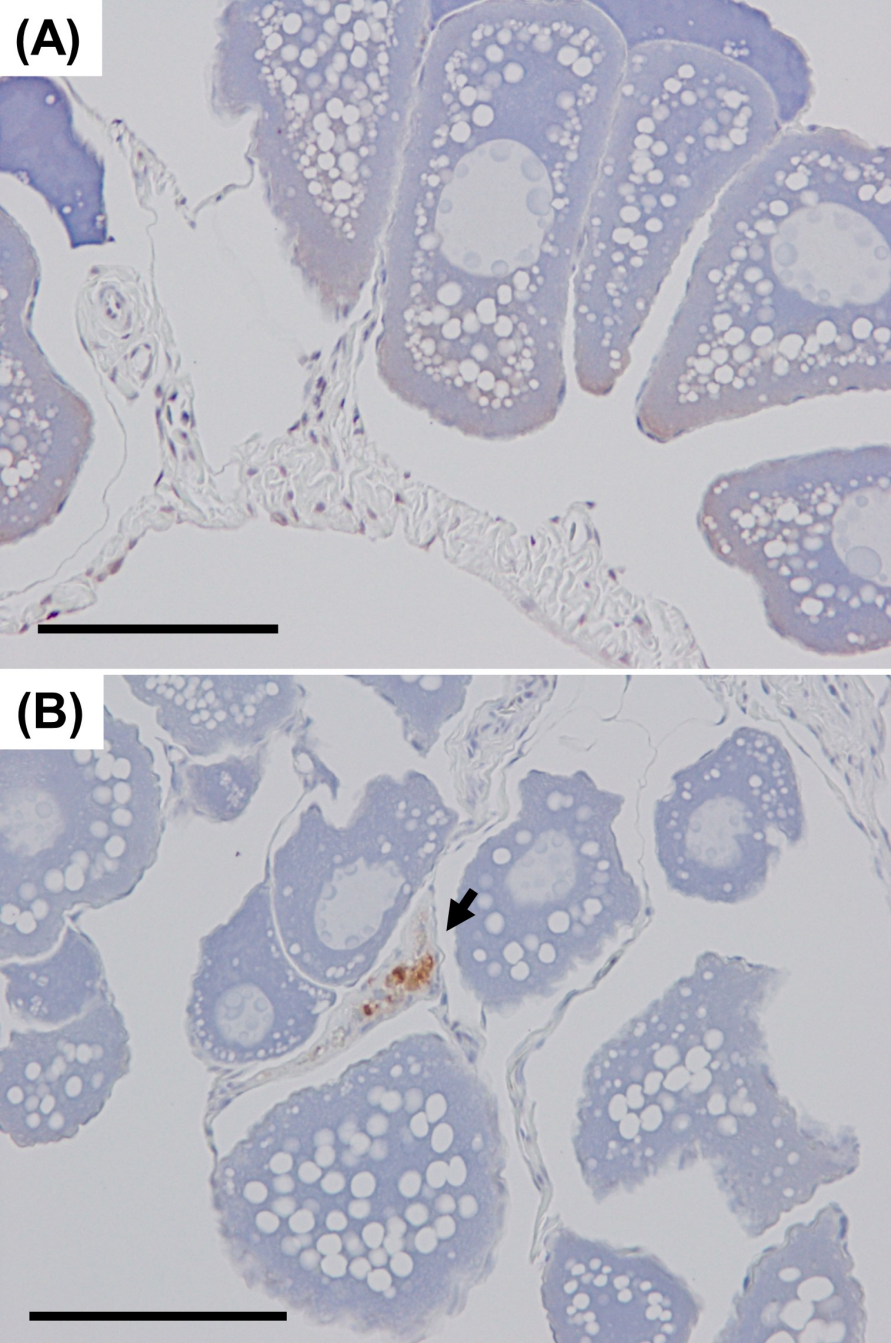
TUNEL assay in female eel ovary samples. (A) wild female eel, (B) cultured female eel. Arrowhead: TUNEL positive cells. Bars, = 100 μm.

Five eel ovarian samples from each eel group were examined. TUNEL-positive cells were only detected in cultured female eel ovaries in interstitial cells (Fig 7). The oocytes in both cultured and wild female eels were mainly in the late-cortical alveoli to early-vitellogenic developmental stage.

## Discussion

In this study, the trans-omics showed clear differences between cultured and wild female eels in their nutritional status just before the beginning of the artificial induction of oocyte maturation. The results also demonstrated these differences influenced the eels’ hormonal status. The findings of this comprehensive study suggested that the main cause of the ovulation problem frequently observed in cultured female eels is their nutritional status.

The transcriptomic analysis revealed that the transcription level of *gh* in the cultured female eel brain was markedly lower than that in wild female eels. In teleosts, growth hormone (GH) affects several aspects of behavior, including appetite, foraging, aggression, and predator avoidance [19–21]; it has also been reported to affect reproduction, including gonad development [22, 23], and steroidogenesis promotion [24]. In humans, GH plays a major role, in conjunction with the gonadotropins, in the control of ovarian function, including puberty, gametogenesis, steroidogenesis, and ovulation [25–27]; however, no data are available on the effect of GH on the ovulation of teleosts. Interestingly, studies of the Asian catfish have demonstrated that an injection of GH in the morning during the early recrudescence phase induced an appreciable increase in ovarian weight and the GSI [28]. In the present study, the ovarian weight and GSI of cultured female eels were found to be lower than those of wild female eels. Our RNA-seq-based transcriptomic analysis data indicate recognizable transcription of *gh receptor 1*, *gh receptor 2*, and *gh-releasing hormone receptor*. However, the transcription levels of gonadotropin receptors were very low. Studies of the Japanese eel have reported changes in gonadotropin receptors in the ovaries during hormone-induced oocyte maturation [4]. During the late vitellogenic stage, transcription levels of *luteinizing hormone receptor* in the ovaries increase. Taken together, these results indicated that compared to low *lh* levels, low *gh* levels mainly influence lower ovarian weight in cultured female eels. Furthermore, it is well known that GH production presents daily rhythms. Although there were no differences between the cultured and wild female eels in the expression levels of circadian rhythms and clock genes, including of period, timeless, clock, aryl hydrocarbon receptor nuclear translocator-like, or melatonin receptors in the brains, further confirmation is necessary to examine whether circadian rhythms in cultured female eels are disturbed.

The metabolomic analysis using GC/MS showed an intense accumulation of glucose-6-phosphate and maltose in the liver of cultured female eels, whereas there was no significant difference in the serum glucose levels between the cultured and wild female eels. Commercial diet for the cultured Japanese eel contains 23%–24% potato starch as a binder (Table 1). In vertebrates, glucose is phosphorylated to glucose-6-phosphate and stored in the liver. The homogenization of eel pancreatic segments shows α-amylase activity [29], indicating that starch is digested to glucose or maltose in the eel’s digestive tract. It has been reported that wild eels in rivers prey on mud shrimp and crayfish [30]. These data suggest that the accumulated glucose-6-phosphate and maltose found in the liver of cultured female eels were mainly derived from starch in the commercial diet and that cultured female eels have a higher intake of carbohydrate than wild female eels. In teleosts, unlike in mammals, amino acids play a more significant role than glucose in insulin secretion [31, 32], and teleosts have generally been considered to be glucose intolerant [33]. However, in angler fish, glucose stimulates insulin and somatostatin-14 production, which suppress GH secretion, and inhibits glucagon secretion [34]. In addition, simultaneously injecting arginine and glucose into European eels, resulted in greater insulin secretion stimulation than injecting either factor alone [35]. The high insulin level probably suppresses the release of glucose and promotes glucose storage. These results indicate that cultured female eels can experience hyperinsulinemia, in addition to significantly high serum triglyceride levels. Studies are needed to measure serum insulin levels in cultured female eels. The commercial diet which contains a high-carbohydrate have been used exclusively in the studies of cultured female eels since the establishment of feminization (by feeding a commercial diet containing estradiol-17β for the first 5–6 months), however, these results also indicated that a high-carbohydrate diet is not suitable for eel broodstock culture.

The transcriptomic analysis also demonstrated that *shbg* transcription levels in the liver were low in cultured female eels. In mammals, it is thought that GH pulsatility and thyroid hormone enhance SHBG transcription *via* hepatocyte nuclear factor-4alpha (HNF4a) [36, 37]. These data suggest that both *gh* transcription and GH secretion are lower in cultured female eels compared to wild female eels, although no significant difference between the two eel groups was observed in the transcription levels of *insulin-like growth factor I* (*igf1*) in the liver. Previously, hepatic IGF1 expression was thought to be fully dependent on GH. However, studies in humans have reported that the total serum IGF1 concentration did not significantly differ between obese and control groups despite GH hyposecretion in obese people [38]. In addition, glucose and insulin can also promote IGF1 expression in fetal rat hepatocytes *in vitro* [39]. These data indicate that GH and IGF1 levels are not always correlated.

Characterization of Shbg in the Japanese eel has previously been reported [40], including a high affinity to E2, androstenediol, and T. In the past, SHBG was thought to regulate the free concentration of the steroids that bind to it, but a recent study found that although cell membranes have a receptor for SHBG, the receptor has not been cloned; the receptor binds unliganded SHBG and the SHBG-receptor complex is activated by an agonist steroid to initiate the generation of cAMP [41]. It has been demonstrated in European eels that there is E2-specific positive feedback with LH expression and T-specific negative feedback with FSH expression during ovarian development [42]. In contrast, the present study found no interaction between serum E2 levels and LH transcription levels or between serum T levels and FSH transcription levels. However, it is possible that Shbg is involved in feedback gonadotropin secretion by steroids. It has also been suggested that in mammals, GH may influence reproductive activity by increasing the secretion of gonadotropin at the hypothalamic and pituitary level and by enhancing the response to gonadotropins at the gonadal level [43]. Confirmation in cultured female eels is needed regarding whether decreased transcription of *gh* and *shbg* levels affects the secretion of gonadotropins through the modulation of steroid signals and gonadal responsiveness to gonadotropin.

In humans, lower GH and SHBG levels are also associated with polycystic ovarian syndrome (PCOS), characterized by menstrual abnormalities, hyperinsulinemia, and hyperandrogenism [44–47]. We therefore, examined whether hyperandrogenic symptoms were exhibited by the cultured female eels. The marker genes, which were selected on the basis of transcriptomic analysis results comparing wild female and wild male brains, initially showed sex-dependent expression. As described earlier, *sl* and *cyp19a1* were selected as highly expressed genes in female eels, and *jumonji* was selected as a highly expressed gene in male eels. Unexpectedly, we did not observe any hyperandrogenic symptoms resulting from the low *shbg* levels in cultured female eels. Although the *shbg* transcription levels were lower in the cultured female eels than in the wild female eels, there was no significant difference in serum steroid hormone levels between the two eel groups. However, circulating T levels tended to be lower in the cultured female eels, and the level of the hepatic *akr1d1* mRNA was significantly higher. The steroid 5β-reductase Akr1d1, is responsible for the conversion of T to 5β-dihydrotestosterone [48]. This is not believed to be an active androgen [49], and its synthesis from T by Akr1d1 may be a process to eliminate excess T [50]. Taken together, these findings suggest that, in cultured female eels, increased free T undergoes clearance in the liver through sequential reduction by Akr1d, and it is possible that up-regulation of *akr1d1* prevented hyperandrogenism in the cultured female eels. Several symptoms in the cultured female eels, other than hyperandrogenism, showed a similarity to human PCOS, such as the lower *gh* and *shbg* transcription levels and ovulation problems. Further studies are required to investigate the effects of a high-carbohydrate diet on the transcription of *gh* and *shbg* and serum insulin levels during artificial oocyte maturation to confirm whether fish, like mammals, can develop PCOS.

Serum 11-KT, a major androgen in the Japanese eel, was slightly but not significantly higher in the wild female eels. The transcription level of *11β hydroxysteroid dehydrogenase short form* (*11β-hsdsf*), which catalyzes 11β-hydroxytestosterone to 11-KT and cortisol to cortisone [51], was also higher in the liver of the wild female eels than in those of the cultured female eels. Thus, high serum 11-KT levels in wild female eels may be associated with higher *11β-hsdsf* transcription in the liver.

Although hyperandrogenic symptoms were not observed in the cultured female eels, there were significant sex differences in gene expression in their brains. As expected, the expression level of *cyp19a1* (also called *aromatase*), was higher in female eel; however, it was unclear why the levels of *sl* were also higher in the female eels. Several studies of the Japanese eel have reported that the number of anti-Sl-positive cells in the pituitary gradually increased during artificial oocyte maturation until the late vitellogenic stage, and then decreased during the migratory nucleus stage [52]. It has also been shown that the relative mRNA levels of *sl* in the pituitary of blue gourami (*Trichogaster trichopterus*) gradually decreased during oocyte maturation [53]. These findings suggest that Sl plays a role in oocyte maturation. Many JUMONJI family proteins have domains, such as ARID, jmjC, and jmjN, that are involved in DNA binding, chromatin binding, and transcription, which suggests that JUMONJI family proteins regulate transcription or chromatin function or both [54]. The transcriptomic analysis in the present study showed that mRNA expression levels of the four *jumonji* family genes were higher in the brain of the male eels. All four genes contained ARID, jmjC, and/or jmjN domains. Interestingly, studies using human cell lines have demonstrated that JMDM2A, a jmjC-containing histone H3 lysine 9 demethylase, interacts directly with the androgen receptor and is used in a hormone-dependent manner in androgen receptor target genes in order to mediate transcription activation [55]. These findings suggest that *jumonji* family genes may contribute to sex differences in the brains of the Japanese eels.

The transcriptomic analysis also showed that apoptosis-associated genes, such as *small nuclear ribonucleoprotein F*, *tumor necrosis factor receptor superfamily member 19*, *cytochrome c*, and *granzyme K*, were highly expressed in the ovaries of the cultured female eels and that *cytochrome c* was highly expressed in the livers [56, 57]. TUNEL assay found apoptotic cells only in interstitial cells of the ovary of the cultured female eels. These data suggest that the caspase-independent pathway of programmed cell death occurs in ovaries of the cultured female eels. Many reports have shown that GH suppresses apoptosis [58–60]. Taken together, these findings indicated that impaired GH secretion is likely to induce apoptosis in the ovaries, and probably the liver, of cultured female eels. Steroid- producing cells (SPCs) have been observed in the interstitial area of fish ovaries and SPCs migrate into thecal layer enclosing vitellogenic oocytes [61]. It is well known that apoptosis in theca-interstitial cells mediates post-ovulatory follicular absorption [62–64], however, it is still unclear whether apoptosis mediates ovarian follicular atresia, which is common phenomenon in teleosts, during pre-spawning period [62, 65]. These data indicated that increased apoptotic cells in interstitial area may affect steroid production, follicular growth and development, and oocyte maturation in cultured female eels.

Trans-omics analysis reveals “the big picture” and provides other insights. In this study, α-tocopherol accumulation was found in the livers, ovaries, and serum of cultured female eels. Previous studies have reported that excess α-tocopherol induces tocopherol radicals and oxidizes ascorbic acid to reduce oxidized tocopherol [66]. Oxidized ascorbic acid, also called dehydroascorbic acid, is degraded to threonic acid and oxalic acid [67]. In cultured female eels, the metabolomic analysis showed high levels of oxalic acid in the ovaries and high levels of oxalic acid and threonic acid in the serum. These data indicate that the cultured female eels had consumed excessive amount of α-tocopherol. The metabolomic analysis also revealed differences between cultured and wild female eels in the DHA/EPA/ARA ratio in all the examined samples. The cultured female eels in this study were fed a diet supplemented with cod liver oil (Table 1). This contains greater amount of DHA and EPA than of ARA [68], suggesting that the dietary DHA/EPA/ARA ratio affected the tissue DHA/EPA/ARA ratio in these eels. It has been reported that eggs from cultured Japanese eels have lower ARA levels than those from wild eels [69]. In addition, several studies have reported that the DHA/EPA/ARA ratio in fish ovaries or eggs strongly influenced the quality of the eggs and larvae [70–72]. ARA is the precursor to biologically active eicosanoids, such as prostaglandins, which are involved in many aspects of reproduction in teleosts including reproductive behavior and ovulation [73, 74]. EPA and ARA compete for the enzymatic pathways involved in the synthesis of eicosanoids; further studies are therefore required to investigate the optimal dietary DHA/EPA/ARA ratio for reproductive performance.

## Conclusion

In conclusion, that trans-omics analysis indicated that the ovulation problem, often observed in cultured female eels may be the result of a high-carbohydrate diet and/or a suboptimal DHA/EPA/ARA ratio in their diet. Interactions between nutrition and reproductive performance have been reported in domesticated animals [75–77], and poor reproductive performance has been reported in cultured broodstock compared to wild fish for several fish species [78–80]. The commercial diet for fish is generally designed to promote weight gain and/or fleshing out, regardless of the fecundity of the fish, and an optimized diet for broodstock culture is not commercially available. Future studies should therefore investigate more extensively the effect of diet on the reproductive performance of fish. The findings of the present study suggested several future experimental directions for the improvement of seed production efficiency, including establishing the effects of a high-carbohydrate diet on GH secretion, the effects of impaired GH secretion on the responsiveness to gonadotropins, the effects of lower Shbg levels on oocyte maturation, and the optimal dietary DHA/EPA/ARA ratios. The comprehensive data obtained in this study also demonstrated the usefulness of the omics approaches for understanding the functionality of diet components in aquaculture.

## Supporting information

S1 Table Primer and probe sequences for RT-qPCR, and PCR efficiencies. Primer and probe sequences for RT-qPCR in this research, and PCR efficiencies (%).
(DOCX)

S2 Table Comparison of gene expression in the livers. List of the significant differentially expressed genes in wild and cultured female eel liver obtained from our RNA-seq data.
(XLSX)

S3 Table Comparison of gene expression in the ovaries. List of significant differentially expressed genes in the ovaries of wild and cultured female eels.
(XLSX)

S4 Table Comparison of gene expression in the brains between wild female eel and cultured female eel. List of significant differentially expressed genes in wild female eel brain and cultured female eel brain.
(XLSX)

S5 Table Comparison of gene expression in the brains between wild eel female and wild male eel. List of significant differentially expressed genes in the brains of wild female and male eels.
(XLSX)
