# Supplementary material for: Trans-omics analyses revealed differences in hormonal and nutritional status between wild and cultured female Japanese eel (Anguilla japonica)
Source: PLoS One. 2019 May 9;14(5):e0209063. doi: 10.1371/journal.pone.0209063 (PMC6508692; doi:10.1371/journal.pone.0209063)
Supplement: S1 Table — Primer and probe sequences for RT-qPCR in this research, and PCR efficiencies (%). (DOCX) [file pone.0209063.s001.docx]

**Supplemental data**

**Supplemental Table S1.** Primer and probe sequences for RT-qPCR, and PCR efficiencies

| Gene (abbreviation)/Accession No. | Forward primer/Taqman probe/Reverse primer | PCR efficiency (%) | |
| --- | --- | --- | --- |
| 60S acidic ribosomal protein P0 (*rplp0*)/ | 5' CCATTGAAATCTTGAGCGATGTG | Brain | Liver |
|  | 5' ATTAAGACCGGAGACAAGGTGGGC | 95.7 | 97.9 |
|  | 5' AACATCTCGCCCTTCTCCTA |  |  |
| 3-oxo-5-beta-steroid 4-dehydrogenase (*akr1d1*) | 5' CTGGACTACGTGGATCTCTACA | 89.7 | |
|  | 5' TCATCGAGCTGCCCATTGCCTTTA |  |  |
|  | 5' CATTTATCACGAGACGGACCTC |  |  |
| cytochrome P450 1A9 (*cyp1a9*)/ AB020414 | 5' CCTTCCTGGAGGCTTTCATAAC | 85.1 | |
|  | 5' GTCTTCCGCCACTCTTCCTTCGTG |  |  |
|  | 5' TTCTTCATCCCAAAGGACACC |  |  |
| sex hormone-binding globulin (*shbg*) | 5' CTGCACACCAATCCAGAAGA | 99.2 | |
|  | 5' TGCGTCTCTCACTTGGTGGAATCC |  |  |
|  | 5' TAAATCCGCTTCGGACAGAG |  |  |
| follicle stimulating hormone (*fsh*) | 5' TGTGCTTCACCCAGGATTC | 92.6 | |
|  | 5' GGCCTGCAACTTCAGGGATGTAGT |  |  |
|  | 5' TATGAGACTGTGCACTTGCC |  |  |
| luteinizing hormone (*lh*) | 5' TTCTGTGGAGAAAGATGGATGTC | 90.6 | |
|  | 5' TCCAAACATCCATCTGCAGTGGTCA |  |  |
|  | 5' CAAGGACCCAAGCTACAAGAG |  |  |
| growth hormone (*gh*) | 5' GTCATGGGTGTATCCTCTGAAG | 88.8 | |
|  | 5' AGCCTGATGTTTGGGACCTCTGAT |  |  |
|  | 5' GATAAGCTGGAGGACCTGAAC |  |  |
| somatolactin (*sl*) | 5' CACGATGGATCTCTACGACAAC | 91.6 | |
|  | 5' TCCACCAAGCTGATGAATCTGGAGC |  |  |
|  | 5' GTGACCGTCCTCATCAGAAAG |  |  |
| cytochrome P450 19A1 (*cyp19a1*)/ AY540622 | 5' CCAGAGGTAGAGCAGGAAATAC | 101.1 | |
|  | 5' CTGGACACTGTGATCGGTGACAA |  |  |
|  | 5' CTATCATCCAGTGGTGGACTTC |  |  |
| protein jumonji (*jumonji*) | 5' TCACCAGACAAGCACACTAC | 100.5 | |
|  | 5' AAACACTCCTCTACCTCAAACGGCG |  |  |
|  | 5' AGACCAGCAGAAGCTAAAGCCGAGAAGG |  |  |
